# Transactions of the Provincial Medical and Surgical Association

**Published:** 1840-10

**Authors:** 


### Art. VIII.

*Transactions of the Provincial Medical and Surgical Association.* Vol. VIII.—*Worcester*, 1840. *Plates.* 8vo, pp. xcvi. 435.

Were it necessary to enhance the advantages resulting from mutual intercourse and combined effort, the published transactions of our various literary, scientific, and medical institutions would form an admirable series of records to which we might apply for illustration and proof. The volumes which from time to time have emanated from these institutions embody the results of the united labours of the most acute philosophers, and contain numerous essays and papers unrivalled for research and the exhibition of mental power. Such of them as are in a more especial manner devoted to medicine, form a valuable record of much that has been effected in like manner and by like means in the cultivation and promotion of medical science and medical practice. Among these the publications of the Provincial Medical Association are characterized by the attention paid to the subjects of medical topography and medical statistics; and we have no hesitation in saying, that from a judicious perseverance in the same course the most beneficial results must ultimately ensue. We may thus hope to arrive at important facts in respect to the causation of disease, and the effects of physical agents in favouring or impeding morbid actions of various kinds, to which we could not attain by any other means. The practical bearing of the knowledge thus acquired is manifest, and the advantages to be derived by the community from pointing out, not only the causes of disease, but the various methods of isolating and removing these causes, or of avoiding and counteracting their operation, can scarcely be estimated too highly. The volume which has been recently issued by this Association affords further illustration of these principles. The advantages of a combination of numerous individuals in the collection of facts are strikingly evidenced by the report on vaccination, which, although defective in some points, is yet a valuable document, and contains much information which we should in vain look for elsewhere. To this, however, we have before had occasion to advert when noticing it in its separate form. It is to the remainder of the volume, comprising the retrospective addresses of Dr. Symonds and Mr. James, and the elaborate observations and experiments on the variolæ vaccinæ by Mr. Ceely, that we are now to direct our attention.

The Address delivered by Dr. Symonds at the Liverpool meeting is an excellent digest of the progress made in the various branches of medicine during the year preceding its delivery; and whether regarded as a record of medical science, or as a literary composition, falls short in no respect of any of its predecessors. A production of this description is obviously not fitted for extended notice, and we can only therefore refer to it as containing, amongst other subjects, notices of the recent researches on the structure of the teeth, by Owen, Nasmyth, Goodsir, and others; on the epidermoid tissues, by M. Flourens and Dr. Henle; of the experiments of Dr. Reid and of MM. Jules Guyot and Casalis, on the functions of the glosso-pharyngeal and other nerves; of the investigations of Drs. Mandl and Henle, Mr. Gulliver and Dr. Golding Bird, on the formation and composition of mucus and pus; of those of Professor Schultz, Dr.

Mandl, MM. Dubois (d'Amiens) and Denis, Dr. Davy, and others, on the blood; of the highly curious observations of M. Turpin on fermentation, &c. There is little of interest in the department of pathological anatomy, but in that of practical medicine attention is directed to the opinions of Dr. Stokes as to the connexion of dysphagia as a symptom with inflammatory affections of the heart, and the indications afforded by the state of the heart for the exhibition of wine in fever: a notice is also given of M. Woillez' observations on the measurement of the chest as a means of diagnosis subsidiary to percussion and auscultation in pulmonary disorders. The principal topics alluded to in forensic medicine are the experiments of Orfila on the detection of poisoning (recent) by the salts of lead, and M. Devergie's proposal of the detection of spermatic animalcules in the urethra as a new test of death by hanging. This test has however since been found inapplicable in consequence of the animalcules having been ascertained to be present under other circumstances. In giving an account of the Army Statistical Reports of Mr. Marshall and Major Tulloch, of M. Quetelet's Essay on the Influence of Season on Mortality, and of Dr. Clendinning's Statistical Researches in relation to Diseased Heart, some excellent observations are made on the value of the numerical method, and the precautions necessary in its employment as a means of directing our choice of remedial measures. The following remarks are so just and apposite that we cannot refrain from quoting them:

"Allowing, then," says Dr. Symonds, "that the value of a remedy has been established by the numerical method, can we therefore apply it to any given case? Or to put the question in another form: it has been proved that eight out of ten cases similar to that before us have been cured by an antiphlogistic treatment, but only six out of ten by stimulants; treated by the one method, the patient's chances are eight to two, by the other only six to four: shall we, therefore, adopt the former? *In the absence of any other reason* for determining the choice, we might be glad to lay hold of one so apparently sound and palpable.

"It is this kind of reason, though not numerically expressed, which leads the routine practitioner to abbreviate his labour. It would be sufficient for a hospital physician, who (if such there be) aims at exhibiting a certain proportion of cures to the cases treated, without caring about the fortunate or unfortunate *individuals.* It would answer to a military practitioner, whose general might say to him, 'Go into yonder sick tents and cure me a certain per centage, I cannot do with less in the approaching action.' But it would not be sufficient for him who regards his patient, not as a mere unit in a future table, but as a *man,*—a being, the current of whose joyous feelings, and ennobling thoughts, and benevolent exertions, may be turned into the dark chasm of death, or hold on its glorious way, mainly through *his* interference; a man whose loss would make a blank, not merely in a column of figures, but in a loving household, an admiring circle of friends, nay in a whole community. The *individual* then must be considered; and because, as an *individual,* he has a peculiarity of organization, which, when deranged, ought to receive a peculiarity of treatment, we must consider anxiously, and we may often, very often, succeed in ascertaining the manner in which his idiosyncracy will modify the operation of remedies, which in the majority produce a different result. We shall use the numerical method just as that of ordinary experience; it will *suggest,* not *determine,* the choice of our remedies." (pp. 173-4.)

The concluding portion of the address is occupied with a notice of the principal works published during the year; some remarks upon the recent regulations of the College of Physicians, in the praises bestowed on which we do not by any means agree, and a brief but characteristic memorial of the brilliant and eccentric career of Broussais.

The Retrospective Address on Surgery delivered by Mr. James, though deficient in method and arrangement, is not less valuable in affording a detailed account of the late improvements and acquisitions to our knowledge in this department. It treats in succession of hernia, hydrocele, lithotomy and lithotrity, surgical diseases of the vascular system, fractures, luxations, anchylosis, contractions, excision of the joints, amputation, diseases of the eye and of the ear, tetanus, bronchotomy, necrosis, the teeth, syphilis, glanders, diseases of the rectum, inflammation, and obstetrics. Upon each of these subjects much valuable information is collected, and the remarks on fractures are illustrated by two plates descriptive of an ingenious apparatus for keeping up graduated and regular extension. Mr. James, it will be observed, differs from Dr. Macartney upon the question as to the effects of the process of inflammation in the reparation of injured parts, and adheres to the Hunterian doctrines:

"It will perhaps be ever very difficult," he observes, "to determine the exact point below which, if I may be so allowed to express myself, the actions or the efforts of an injured part are directly and entirely reparative, and above which they tend, in very various degrees, either to a more indirect mode of reparation, or on the contrary to destroy that which they cannot repair; still, with respect to the question, whether these are or are not modifications of the same actions, I must with great deference say that my own opinion accords with that ascribed to Mr. Hunter. If this question were confined to the process of the reparation of wounds by the first intention, it would not have been very material; but if the doctrine leads further, as it really does, and we are rather to regard inflammation as the opposite in all cases of the reparative process, it must be viewed with great distrust, and the many facts which tend to establish a contrary opinion ought to be well weighed before it is received." (pp. 247-8.)

The following remarks upon the treatment of inflammation deserve the attentive consideration of those whose opportunities of practice or clinical instruction have not been sufficiently extensive to lead them to discard the theoretical and partial views too often adopted from mere reading or oral teaching:

"Experience has now amply shown, that any preconceived opinions as to the nature of inflammation generally, which blindly lead to the adoption of a depleting or lowering plan of treatment in *all* cases are erroneous. Inflammation, to be studied with success, must be observed in its different varieties. I feel warranted in stating this, when it is in my power to say that bleeding and other antiphlogistics have been fully acknowledged to be injurious in *some* forms of acute inflammation. I may mention purulent ophthalmia in particular: that, on the contrary, wine has been resorted to with advantage in the earliest stages of others, as in certain varieties of erysipelas; and that the most severe kinds of peritonitis that can be imagined, namely, that produced by the escape of the contents of the bowels from wound or rupture, are treated with the greatest chance of success by large doses of opium, a practice first recommended by Dr. Stokes, and borne out by two very remarkable cases related by our able associate Mr. Toogood. I may add that M. Malgaigne has insisted much on the advantage of treating inflammation arising from operations or injury by large and repeated doses of this important remedy; and if it is allowed that the excess of inflammatory action is commonly due to an excessive sympathy, we shall be at no loss to understand how a remedy so admirably adapted to control this should prove so highly beneficial. In many cases the best antiphlogistic is support adequate to the occasion." (pp. 254-5.)

Towards the conclusion of the address we find, as in that of Dr. Symonds, a brief consideration of the merits of the numerical system, some remarks on the working of the Anatomy Act in country districts, and a short record of Mr. Thomas Blizard and Mr. Henry Earle, both of whom have recently been lost to the profession.

Mr. Ceely's Observations on the Variolæ Vaccinæ, together with his experiments upon the vaccination, retrovaccination, and variolation of cows, with which the volume concludes, are of the highest degree of interest and importance. They need no prefatory encomium, and we feel that we shall best discharge our duty to our readers by at once proceeding to give a careful analysis of this very excellent paper.

Led by doubts upon some of the Jennerian doctrines, and at the same time prompted by the direct application of Mr. Dodd, one of the secretaries of the vaccine section of the Association, Mr. Ceely commenced a series of experiments which he had long contemplated. These were carried on in the Vale of Aylesbury, of the topography of which, its general features, geological characters, &c. he gives a condensed account. Bronchocele and struma, in all its varied forms, are, it appears, endemic in this rich and fertile country, and dyspepsia and neurotic diseases very prevalent. The epidemics are pretty much the same as those in other places exposed to the influences of malaria—fevers, dysenteric and acute gastro-intestinal affections, &c. The sheep in wet seasons, together with the hares and rabbits, suffer extensively from liver-rot, while dry cows and even young heifers are liable to become affected with the *garget* (inflammation and induration of the udder.) The *aphtha epizootica*, a disease which has lately been prevalent on the continent, has been observed in one dairy. In addition to the variolæ vaccinæ the milch cows are subject to various eruptive diseases and spurious pocks as they are termed; among these are the yellow pock, the bluish or black pock, and the white pock, to which the author purposes calling attention at some future period. The variolæ vaccinæ, which seem to have been long known in this locality, are treated of, 1st, as they appear naturally or are produced casually in the cow by the manipulations of the milkers; 2d, as they are produced by vaccination; 3d, by retrovaccination; 4th, by variolation.

1. *Casual Variolæ Vaccinæ.* The natural or casual cowpock is said to appear most commonly during the spring, rarely in the heat of summer, though Mr. Ceely has observed the disease at all periods. It is occasionally epizootic, more commonly sporadic, and extremely irregular in the periods of its occurrence, even in dairies situated in the same immediate vicinity, and apparently placed under circumstances in all respects similar. The disease is considered to be peculiar to the milch cow, occurring primarily while the animal is in that condition, and being casually propagated to others by the hands of the milkers. Many intelligent dairymen are inclined to trace its origin to the equine vesicle, but Mr. Ceely has himself never been able to connect it with this source. Fever, or other constitutional symptoms, either in the animals primarily affected, or in those to whom the disease has been conveyed by the milker, are very rarely observed, heat, induration, and tenderness of the udder, being generally the first symptoms noticed. It is seldom that a competent observer has the opportunity of seeing the topical symptoms of the primary disease; but according to the statements of the milkers, irregularity and pimply hardness of the teats and udder, especially about the bases of the former, occur in about three or four days after the heat and tenderness of the parts before mentioned. The pimples on the lighter coloured skins are described as being of a red colour, hard, and about the size of a vetch or pea, subsequently increasing to that of a horse-bean, forming vesications, which are soon broken by the hands of the milkers, becoming extremely tender, and rendering the process of milking a very troublesome operation. The following is a condensed abstract of Mr. Ceely's account of the topical symptoms as they appear in the casual affection. About the fifth day after exposure, in thin-skinned animals with chaps and cracks on the teats, small red rather tender papulæ may by close observation be detected near the udder and on the body of the teats. On the sixth and seventh days, in cows with white clear skins, circumscribed indurations are observed; these are generally of a reddish colour, circular, ovoid, or lozenge-shaped, as large as a vetch or pea, or even larger, and with a central depression, in which is visible a small dirty yellowish-white discoloration surrounding a still darker dot or line. The indurations are often interspersed with minute red papulæ of a darker colour, rather acuminated, and frequently abraded at the summits. Some of the tumours have a slender amber-coloured yellowish-brown or brownish-black central crust, and are less prominent than those without the crusts. The tumours on the teats are generally circular, and some of them occasionally smaller than those on the udder; the circumscribed intumescence and induration are also less apparent and less defined, but the pearly or glistening lustre of their margin and part of their centre is nearly as manifest. On the eighth and ninth days some of the tumours appear with more central depression and more elevation of the margin, which is solid, uniform, tense, and shining, extending to seven or eight lines, of a glistening white pearly or silvery hue, and in the centre of many is observed a bluish or slate-coloured tint. Around the base a narrow pale rose or light damask areola, not more than a line or two in width, is often apparent. In others the central crust has increased, and is yellow, brown, or black; a few appear a little pustular in their centres. A few small conoidal vesicles, from the size of a pin's head to that of a pea, some of which have a slight depression on the apex, also appear to have subsequently risen on the teats; but generally the majority of the tumours on these parts are more or less abraded, and lymph is seen exuding from the centres, with the cuticle loose or partially detached, raw surfaces, brown or black incrustations, and other alterations in their natural characters and appearance. Between the tenth and eleventh days the disease in general reaches its acmé; the tumours are often from eight to ten lines in diameter, the centres and central edges of the intumescent margin are of a deeper blue or slate-colour, and the areola, usually of a pale rose-colour, is seldom more than four or five lines in extent, under which the integuments are deeply indurated. The lymph now becomes abundant, raising the cuticle and forming a globular or conoidal vesicle, or freely flowing out from its rupture. Other tumours have an extended brown or black central crust, either slightly acuminated or depressed; others have become flatter, entirely incrusted and perfectly passive. The tumours on the teats undergo similar changes, but in consequence of being for the most part early abraded, ruptured, and irritated, present considerable modifications in the form and appearance of the crusts, the interfluent vesicles being often denuded of cuticle, raw, swollen, with elevated margins, and discharging blood, lymph, and pus. From this period the induration and intumescence subside, the incrustation and desiccation proceeding until from the twentieth to the twenty-third day, when the crusts spontaneously separate, leaving shallow, smooth, oval, or circular cicatrices of a pale rose, white, or whitish colour. On the teats, however, where the process has been disturbed, large black, solid crusts, often more than an inch or two in length, are to be seen in different parts, some firmly adherent to a hard and elevated base, others partially detached from a raw, red, and bleeding surface; in other parts the surface is denuded, florid, red, ulcerated with small central sloughs, secreting pus and exuding blood, the teats being excessively tender, hot, and swollen. (pp. 307-12.)

The regular duration of the disease would seem to be from twenty to twenty-three days, the eruption appearing about the third or fourth day after exposure, reaching its full development in from six to seven days, and declining in five or six more; the crust separates in about five or six days from its formation, leaving a cicatrix behind.

Various irregularities and anomalies occur in the duration of one or more of the stages of incubation, eruption, decline, and incrustation, &c., in the size, form, colour, and general characters of the vesicles, in the quantity and quality of the lymph, in the colour and extent of the areola, &c., which Mr. Ceely carefully describes, contrasting these variations with the more usual appearance of the disease. Some of these deviations from the regular progress depend upon differences in the thickness of the skin of the animal; others, which are more apparent than real, upon the colour, —the preceding description referring to the disease as it is seen in animals with light skins; others upon peculiarity of constitution, &c.

"An anatomical examination of the structure of the vesicle, just before it attains maturity, shows that its colour, indurated margin, and central depression, depend on the existence of an adventitious membrane formed in the corium, and secreted by the papillæ. It is raised in the form of a zone, and is intimately connected with the epidermis. It has a cellular structure, in which is secreted and contained a clear viscid lymph. The cells appear to be arranged in two concentric rows, and are separated from each other by whitish radiating partitions, which, at their converging extremities, are united by a central membranous band. The dusky central spot which marked the first change of the pimple into the vesicle, and which has now become darker and more distinct, seems to be caused by a greater or less degree of separation and desiccation of the epidermis, stretched over a crypt-like recess, which contains a small quantity of semi-concrete lymph-like matter, occasionally a turbid opaque fluid. This cellular, adventitious, membranous conformation, though differing in extent and amount in different vesicles, is invariably present, and is not less essential than diagnostic.......The lymph within the cells having become more abundant and less viscid, and somewhat opaque, bursts and breaks up the cells and their connecting band, separates the epidermis from its attachment to the subjacent adventitious membrane; and the vesicle, losing its central depression, becomes more or less acuminate, presenting a conoidal or semi-globular form. The lymph soon acquires a pale straw colour, or light amber hue, and speedily becomes more serous, turbid, and opaque." (pp. 317-8.)

We cannot follow these observations further; but the whole account here given of the apparent anomalies and irregularities of genuine cowpock is faithfully drawn up, and the tracing of these out to their respective causes satisfactorily effected. The descriptions are illustrated by two plates.

In connexion with the observations on the natural or casual cowpock in the cow, is an account of the same affection casually induced in man, which will be read with much interest. Mr. Ceely is of opinion, that the casual cowpock, though not so often met with as it was forty or fifty years ago, is yet, notwithstanding the progress which vaccination has made among the peasantry, of more frequent occurrence in the Vale of Aylesbury than in many other parts. Cases, however, of spurious eruptive disease, communicated from the cow and mistaken for genuine cowpock, are yet more frequent, a circumstance which it is of importance for the practitioner residing in similar districts to bear in mind, since by leading to the neglect of correct and efficient vaccination, fatal consequences have ultimately resulted from subsequent exposure to the infection of smallpox. Casual cowpock is not unfrequently met with in persons who have had smallpox or been previously vaccinated, in some also who have before gone through the casual disease. The appearances which the casual cowpock presents in man have been admirably described and delineated by Jenner; but, according to the experience of Mr. Ceely, considerable variety exists in the casual as well as in the inoculated form of the disease, both in the intensity of the constitutional symptoms and in the character of the vesicle, extent of areola, &c.

"Instances are occasionally met with, (arising probably from an insufficient application of lymph, or a local or constitutional indisposition to receive the disease in the usual way,) where the vesicles are imperfectly developed, and there is little or no obvious constitutional disturbance, exhibiting a marked contrast to the local or general effects on other individuals affected at the same time from the same source. I have seen such cases followed by distinct though mild smallpox. From these occurrences, and others presently to be related, I have been accustomed to consider milkers liable to smallpox, contrary to their own expectations: first, from having been the subjects merely of the spurious diseases; secondly, from imperfect vaccination at a late period of the disease, with *deteriorated* and purulent virus, or even with *perfect* lymph, of which they had not at the time been sufficiently susceptible." (p. 336.)

Of the spurious eruptions liable to be confounded with the genuine vaccine, the white pock would seem to lead to the most frequent mistakes. "On very thick skins, about the sixth or seventh day of its existence, it sometimes appears as a raised, circular, well-defined, firm vesicle, with a small violet or pink areola, and a slight central depression, with a light brown discoloration." On close examination, however, it will be found to be neither cellular in its structure, nor possessed of fluid contents.

The mode of procuring primary cowpock lymph, and the precautions necessary to be observed in practising vaccination with lymph of this description, are here carefully described, and the difficulties, frequent failures, and disappointments, which are liable to occur before the virus becomes sufficiently assimilated to the human constitution to ensure its ready transmission from one individual to another, are pointed out. Often in constitutions either naturally insusceptible to or only imperfectly affected by primary lymph, subsequent vaccination with ordinary lymph has succeeded. A similar difficulty was also observed to arise with lymph derived from the vesicles of casual cowpock on the hands of the milkers, and in the earlier removes of lymph taken from the primary vaccinations; even in the most successful, the several stages of the process are commonly more or less protracted, the character of the vesicles, although they are frequently large and well developed, is on the contrary often defective in these points; but, says Mr. Ceely, "they admit of very remarkable improvement by transmission of the lymph through a series of well-selected subjects, by which process also in a very short time most of the defects, and some of the evils, connected with the use of primary lymph may be dissipated, and the lymph rendered milder and more suited to general purposes." (p. 348.)

2. *Vaccination of the cow with primary lymph.* The process followed by Mr. Ceely for the effecting of this object differs from the casual vaccination of the cow by the milkers, in the designed application of the lymph through punctures artificially made on parts selected as being appropriate for its reception. The animals chosen were *sturks* (young heifers), with a view of ascertaining how far other than milch cows were susceptible. Some of these, about ten months old, were vaccinated with lymph taken from a milch cow in the inside of the ear, on the teats, and near the vulva. The punctures in general were early inflamed, but the papular stage was not well marked, and appeared postponed; the vesicles were normal, but declining on the eleventh day. The only constitutional affection observed was a slight acceleration of the pulse. The lymph taken from these vesicles on the tenth day, when transferred to man, produced an affection which differed in no respect from that produced by primary lymph from the milch cow, with the exception that the inflammation and induration at the base of the vesicles were less considerable, and the subsequent scars, though well defined, rather less deep.

3. *Retrovaccination.* In Mr. Ceely's experiments upon retrovaccination, or vaccination of the cow by humanized lymph, performed chiefly upon the sturk, with either dry lymph or lymph preserved in capillary tubes, the success was often very uncertain. Four of these experiments are detailed: the lymph employed was in one of several years' standing; in another, lymph from the Smallpox Hospital, where it had been in use little more than two years; in a third it was variola-vaccine lymph of the nineteenth remove; and in the fourth, retrovaccine lymph of the fifth remove from No. 2. The general results obtained with the lymph thus generated, when transferred again to the human subject, were a retardation in one or more of the stages, and a slight apparent deterioration in the characters of the vesicle produced. It would seem to have suffered a temporary impairment of its activity, rather than to have gained power by the retransmission through the cow, although it should be observed that in the course of a very few removes it regained its former efficiency.

"Lymph which had passed from arm to arm with the greatest promptness and facility, and produced the finest effects in its first removes from the costive vesicle of the retrovaccinated cow, is not so readily absorbed by many individuals. It rarely fails; but papulation is retarded, and though the vesicle may attain maturity at the normal average period, the completion of that stage is frequently postponed. The vesicles are often smaller, and the disease really not so well developed, as by the stock from which it was derived. But these changes do not appear after the second or third remove. The lymph is restored to its former qualities, and produces its former effects. Some of the retro-vaccine lymph of the second experiment, transmitted to the Smallpox and Vaccination Hospital, from whence it was originally derived, was passed through a long series of subjects, under the inspection of Dr. Gregory and Mr. Marson, who, though better able to make the comparison, were unable to detect any sensible difference in its local or constitutional effects after the second remove. On the third remove it became 'very fine,' as it was before its transit through the cow." (p. 368.)

We agree with the author in the inferences which he is disposed to draw from these experiments, (which, as far as they go, confirm the conclusion at which we had before arrived, as to the questionable utility of the proceeding,) compared with the results of his experience in cowpock generally, that "primary vaccine, passed by artificial means through a series of cows, would lose much of its acrimony, and produce on man a milder but abundantly active and characteristic disease; but that humanized vaccine, by a similar process, should ever acquire all the attributes of primary vaccine, or become so much more *brutalized* as to deserve the epithet of 'renewed,' is more than many are prepared to expect." (pp. 368-9.) It is well observed, that the general practice of vaccinators in selecting their lymph from the most perfect and well-developed vesicles in itself tends to support these conclusions. Some excellent remarks upon the question as to the actual degeneration of the vaccine virus follow, in which the effects of warmth of climate, long-continued use, repeated transmissions, &c., are considered. So long as the lymph remains active, producing a well-developed vesicle, regular in its stages, and affording abundance of clear and limpid lymph, it is surely unnecessary to resort to the cow for fresh supplies of the primary virus; much less is it requisite to have recourse to the troublesome and difficult process, at best of doubtful efficacy, of retransmission through that animal:

"But when lymph is found uniformly deficient in infecting property, vesicles abnormally rapid in their course, at their greatest development on the seventh day, yellowish in appearance on the eighth, with turbid lymph, central desiccation on the ninth, and a miserably small crust falling on the fifteenth or eighteenth day, common prudence dictates its discontinuance, and urges the adoption of a new supply, although constitutional symptoms may not be absent, for *weak* lymph may not be better than *late* lymph." (p. 376.)

Six plates of the retrovaccination of the cow are given in illustration of the examples adduced.

4. *Variolation of the cow.* In consequence of the doubts thrown upon Dr. Sonderland's experiments, and the inferences which he had deduced from them, Mr. Ceely resolved to investigate anew the alleged origin of cowpock from man, and the correctness of the opinion as to the rarity of the disease of late years in the cow. After careful and extensive enquiry, the comparative rarity of cowpock was ascertained, and the disease was found to occur under the following conditions: first, during epidemic smallpox, no cases of variola being known in the neighbourhood; secondly, when variola was prevalent in neighbouring towns or contiguous villages; thirdly, when cases of smallpox occurred in the same village with the affected farms. Mr. Ceely has, however, never succeeded in tracing out positive or probable intercourse between the affected cows and persons labouring under smallpox, except in one instance, in itself perhaps not entitled to much weight. The rarity of the cowpock in the cow, attributed by Dr. Sonderland to the diminished frequency of variola since the introduction of vaccine inoculation, having been ascertained, and in the cases which occurred no direct evidence having been obtained as to the dependence of the one disease upon the other, Mr. Ceely determined to put the matter to the test of experiment. In the first instance Dr. Sonderland's method of covering the cows with infected clothing was had recourse to, but the results were altogether inconclusive, no effect having been produced either from the experiments on the one hand, or from the subsequent inoculation of the animals with smallpox virus, with the vaccine, or with retrovaccine matter on the other. Mr. Ceely then undertook a series of experiments upon variolous inoculation, subjecting several young heifers to this process. To these we have before had occasion to refer in noticing the report of the vaccination section. (Br. and For. Med. Rev Vol. IX. p. 81.) We shall here quote the second experiment of the series as being not open to the objections which might be alleged against the first, the progress of which was interfered with by subsequent vaccination.

" *Experiment second.* White sturk, thin skin, [which had been inoculated with smallpox matter fourteen days previously without effect,] February 15th, reinoculated with smallpox virus of the seventh and eighth day (variola discreta) on the left side of the vulva and under fold thereof, as before, and near the verge of the anus. Virus liquid, some pellucid, some opaque and puriform, in capillary tubes, was forced into eight punctures, which were deluged with it; the punctures being afterwards irritated with points deeply charged with the same, which were suffered to remain in the punctures. Third day, nothing remarkable. Fifth day, four upper punctures near the verge of the anus enlarged and elevated; four on the under fold of the labium, elevated and red, but less enlarged. Sixth day, all present the appearance of the vaccine vesicle. The four upper are larger, but seem only tubercular; four lower on the under fold of the labium have a deep damask hue, and appear like oval or circular solid elevated rings, with central depressions; from one of these took clear lymph with much difficulty, and scantily charged thirty-nine points. Seventh day, upper tubercles seem diminishing, lower vesicles seem flatter but broader. Eighth day, four upper still appear tubercular, of a lighter colour than their ground; four lower vesicles rather augmented, have a light damask hue. Took lymph again from one of them; the other three, not readily yielding lymph on a careful puncturing, were not further disturbed, from a desire to witness their progress, slight central crusts. Ninth day, the four vesicles enlarging; again opened the inner margin under the daily increasing central crust of the vesicle first opened, and charged twenty points; tubercles diminishing. Tenth day, one of the tubercles rather larger; four lower vesicles increasing. Charged twenty-seven points. Vesicles have a bluish, reddish, glistening appearance; two of them rather red at the base; one or two rather raw on each side. Eleventh day, brown crusts over the centre of the vesicles, which appear declining. Twelfth day, declining, with increasing crusts of a blackish brown colour, within a slightly-elevated margin. *Vaccinated* in several punctures on the margin of the right labium with many points, well charged with sixth-day retrovaccine lymph; two removes. Punctures slightly tumid for a day or two, but quickly subsided. No result. March 12th, four scars as large as peas, in the situation of the four vesicles, depressed, pale rose colour. *Reinoculated* with smallpox virus (confluent), fifth day, in four punctures, on inside of left labium. *Revaccinated* in three punctures, many points, of fifth and seventh day lymph from a child. No result." (pp. 385-7.)

The third experiment was a failure. Every precaution to avoid error seems to have been adopted in these experiments; the smallpox virus was taken by Mr. Ceely himself on newly-prepared points which had never been used before; and for the second experiment new capillary tubes were charged: the subjects from which the virus was taken were healthy young men, the pocks being remarkably fine, large, plump, and numerous. The first experiment which, in consequence of vaccination having been performed on the ninth day of variolation presents, as Mr. Ceely observes, the application of Bryce's test in the cow, is illustrated by six beautiful coloured plates, in which the simultaneous progress of the variolous vesicle, and of the subsequent vaccine vesicles rapidly overtaking it is faithfully delineated. The drawings are taken on the tenth, eleventh, twelfth, thirteenth, fifteenth, and sixteenth days of variolation, corresponding with the second, third, fourth, fifth, seventh, and eighth of vaccination, the whole of the vaccine vesicles as well as the variolous one being at their height on the fifteenth day of variolation, and manifestly on the decline on the sixteenth. The second experiment is illustrated by seven plates, representing the appearances respectively observed on the fifth, sixth, eighth, ninth, tenth, twelfth, and twenty-sixth days, the last plate showing the smooth pale marks left after the fall of the crusts. Both animals were reinoculated and revaccinated without effect. No indisposition was observed during the progress of the disease; and increased heat and redness of the mucous membrane of the vulva with slight acceleration of the pulse were the only symptoms noticed.

Mr. Ceely states it as his opinion, that were we able to manipulate the animal with variolous matter in the same manner as the best vaccinators of the cow (the milkers) do involuntarily with cowpock matter, we might expect a larger measure of success; but as this is manifestly not attainable, he throws out some valuable suggestions as to the selection of the subject, the season of the year, and the mode of operating, best fitted to promote success in like experiments. The teats and udder of a milch cow, having a thin skin, and not too old, should be chosen for the operation, and care taken to ascertain that the animal has not been previously the subject of the vaccine; the back part of the udder or base of the teats are the most preferable situations, as being more out of the way of the milker; if a sturk or young bull be employed, then the lip of the vulva or the scrotum are the best parts. The skin should be freely incised, though superficial wounds of long standing, in imitation of the chaps and fissures on the teats, which favour the reception of the casual vaccine, would probably answer well. Liquid smallpox lymph of the fifth or sixth day should be used abundantly in preference to points. Extreme heat and extreme cold would seem to be alike unfavorable, while on the contrary a warm, close, moist atmosphere, such as it is well known is favorable to the propagation and malignancy of smallpox, would probably be advantageous. "Many punctures, much lymph, every natural advantage, and all the resources of art, seem necessary to ensure success to a moderate extent." (pp. 399-402.)

Mr. Ceely then proceeds to relate the effects of the lymph, illustrating his remarks by examples and plates. In the first example given, his assistant, Mr. Taylor, had accidentally inoculated himself from the matter of the single variolous vesicle developed in the first experiment on the cow; he had been previously vaccinated in infancy, and had subsequently had modified smallpox; the result was modified vaccine with a general eruption of roseola and vesicular or vaccine lichen. Lymph taken on the tenth day from the variolous vesicle referred to, was introduced into the left arm of five children, four punctures in each; in two of the children two vesicles were produced, in two others one only, and in one the punctures all failed. The vesicles in two especially were very fine, with full areola; the primary constitutional symptoms were slight in all; the secondary symptoms were proportionate to the extent and character of the areola. One child, Joseph Woodbridge, in whom the areola was very extensive, suffered severely, and had roseola with vomiting and delirium. Lymph of the fifth day from the vaccine vesicle of the same animal, together with lymph of the tenth day from the variolous vesicle, was inserted into two children, three punctures being made with each kind of lymph on each child; in one the retrovaccine lymph failed, while the variolated lymph produced two very fine active vesicles; in the other, both sets of punctures took effect, those with the retrovaccine being more early developed. In the subsequent removes it was impossible to discover any difference between the course and effects of the lymph derived from the variolous vesicle of the tenth day, and that from the retrovaccine vesicles of the fifth or seventh day.

Of the effects of the lymph from the second sturk we shall select Emma Jaycock as an example, since the lymph subsequently kept up by Mr. Ceely was derived from this source. She was fourteen years of age, of dark swarthy complexion, thin skin, and rather florid. Two points of sixth-day primary variola-vaccine lymph, and four of eighth-day lymph were inserted into six punctures. On the fifth day, four of the papulæ had ash-coloured summits, and seemed vesicular, two were doubtful. On the seventh day, there were five small, distinct, reddish gray or ash-coloured vesicles, one very small. On the eighth day, the vesicles were advancing, of unequal size and of irregular form. She had been slightly indisposed on the fifth and sixth days, the axilla was painful on the seventh day, and she was then giddy and sick, but felt worse on this, the eighth day. On the ninth day the areola commenced, and she complained only of headach; on the eleventh it was fully developed, when all her symptoms returned in an aggravated form. On the twelfth day it declined, but the turgid vesicles having burst, renewed inflammation and induration, with circumscribed sloughing and ulceration of the skin ensued, and rather deep scars were left. (pp. 409-10.)

"Her brother, Lewis Jaycock, aged twelve, was vaccinated from her vesicles on the seventh day; he had a dark dusky complexion, and very thin skin, and was not so florid. He exhibited papulæ in every puncture on the fourth day, all of which were vesiculated on the fifth, on the evening of which day his axilla was sore; he felt headach, was giddy and sick. On the sixth day all these symptoms had rather increased, the vesicles were advancing; thirty-eight points were charged, and two children vaccinated from him, and he was re-vaccinated with his own lymph. On the seventh day he was in all respects better; vesicles advancing; took thirty-eight points and vaccinated a child. On the eighth day the areola commenced, and the *test* vesicles were forming. On the ninth, areola pretty extensive, though pale, from the character of his skin, when headach, &c. returned. On the tenth areola increased, but only two vesicles were entire; the rest spontaneously burst, or were rubbed; still complains. On the eleventh day they were nearly all destroyed. Here the vesicles were remarkably small, but not so on the subjects vaccinated from him, on the three children (two infants), all healthy and robust, with thick skins, nothing could be more satisfactory than the character of the vesicles, and the evidence of constitutional affection." (p. 413.)

These cases, as well as those of the third, fifth, and fourteenth removes, are illustrated by plates, showing the progress to have been in the highest degree satisfactory. Mr. Ceely gives the following summary of the development and subsequent course of the vesicles in those who were the subjects of his experimental researches.

"In the majority of instances, in propagating from arm to arm, distinct papulation was apparent on the second day; on the third it was not only visible, but elevated and well defined; on the fifth and sixth, vesiculation was abundantly obvious, and lymph was often taken on those days; on the seventh day vaccination was frequently performed, and points were often charged; on the eighth the vesicle commonly exhibited a bold, firm, and glistening aspect; between this period and the ninth day the areola generally commenced; (but in young infants, with tense and sanguine skins, it appeared *early* on the eighth;) by the tenth day the vesicle was commonly in its greatest beauty and highest brilliancy, glistening with the lustre of silver or pearl, having the translucency and appearance of crystal, or shining with a pale blue tint, occasionally of a dull white or cream colour, bold and elevated, with a narrow centre and broad margin, or flat and broad in the centre with an acute margin, occasionally not raised above the level of the skin; on this and the eleventh day an extended, and generally vivid areola existed, with more or less tension and induration on the integuments; at this time the lymph was frequently pellucid, and often perfectly efficient. From the eleventh to the thirteenth day gradually increasing; in many individuals, both children and adults, sometimes the entire vesicle, at others only the central parts, reflected a blue or slate-coloured tint from the congested or ecchymosed subjacent adventitious structures, proportioned to the texture and degree of translucency yielded by its desiccated epidermis. On the thirteenth and fourteenth day, particularly on clear skins, moderately thick, the vesicles attained a considerable size, measuring often in their longest diameters six and a half or seven lines, and acquired a light-brown centre, from commencing desiccation, which was surrounded with an outer margin of dull white, or pale dirty yellow, soft and flaccid, and still possessing fluid contents. During this period the areola, of a dull red or damask hue, would revive and decline again and again, and even to the sixteenth or eighteenth day, the period to which complete desiccation was frequently protracted. The crust commonly partook of the form of the vesicle; it was often prominent and bold; varying in colour from that of a chesnut to that of a tamarind stone. It fell generally about the twenty-third or twenty-fifth day, often later....leaving cicatrices of variable depth and extent." (pp. 414-16.)

In none of the adults, with the exception of Mr. Taylor's case, was there any attendant eruption, nor in any child the slightest approach to a varioloid character; roseola, strophulus, and lichen being the principal eruptions noticed to accompany the development of the vesicle in children.

We have thus endeavoured to give an analysis, or rather a condensed abstract of the contents of this important paper; and have only to remark, in conclusion, that the highest credit is due to Mr. Ceely for the care and precaution with which his experiments were performed, for accuracy and fidelity in detailing the facts observed, and for the admirable manner in which they are illustrated and commented upon throughout. In every future history of vaccination the name of Mr. Ceely will be recorded with the honour due to that of an improver of medical science, and a benefactor of his fellow-men.

